# Mechanistic insights into the role of probiotics in modulating immune cells in ulcerative colitis

**DOI:** 10.1002/iid3.1045

**Published:** 2023-10-13

**Authors:** Ni Guo, Lu‐lu Lv

**Affiliations:** ^1^ Department of Gastroenterology Shengzhou People's Hospital (The First Affiliated Hospital of Zhejiang University Shengzhou Branch) Shengzhou Zhejiang Province China

**Keywords:** gut microbiota, immune cells, inflammatory cytokines, probiotics, ulcerative colitis

## Abstract

**Background:**

Ulcerative colitis (UC) is a persistent inflammatory disorder that affects the gastrointestinal tract, mainly the colon, which is defined by inflammatory responses and the formation of ulcers. Probiotics have been shown to directly impact various immune cells, including dendritic cells (DCs), macrophages, natural killer (NK) cells, and T and B cells. By interacting with cell surface receptors, they regulate immune cell activity, produce metabolites that influence immune responses, and control the release of cytokines and chemokines.

**Methods:**

This article is a comprehensive review wherein we conducted an exhaustive search across published literature, utilizing reputable databases like PubMed and Web of Science. Our focus centered on pertinent keywords, such as “UC,” ‘DSS,” “TNBS,” “immune cells,” and “inflammatory cytokines,” to compile the most current insights regarding the therapeutic potential of probiotics in managing UC.

**Results:**

This overview aims to provide readers with a comprehensive understanding of the effects of probiotics on immune cells in relation to UC. Probiotics have a crucial role in promoting the proliferation of regulatory T cells (Tregs), which are necessary for preserving immunological homeostasis and regulating inflammatory responses. They also decrease the activation of pro‐inflammatory cells like T helper 1 (Th1) and Th17 cells, contributing to UC development. Thus, probiotics significantly impact both direct and indirect pathways of immune cell regulation in UC, promoting Treg differentiation, inhibiting pro‐inflammatory cell activation, and regulating cytokine and chemokine release.

**Conclusion:**

Probiotics demonstrate significant potential in modulating the immune reactions in UC. Their capacity to modulate different immune cells and inflammation‐related processes makes them a promising therapeutic approach for managing UC. However, further studies are warranted to optimize their use and fully elucidate the molecular mechanisms underlying their beneficial effects in UC treatment.

## Graphical abstract

This comprehensive review explores the therapeutic potential of probiotics in ulcerative colitis (UC) by examining their impact on immune cells. Through an exhaustive search of relevant literature, the review highlights how probiotics interact with immune cells, promote regulatory T cell growth, inhibit pro‐inflammatory cell activation, and regulate cytokine and chemokine release, all contributing to the management of UC. While probiotics show promise in immune regulation for UC treatment, further research is needed to optimize their use and better understand the underlying molecular mechanisms.

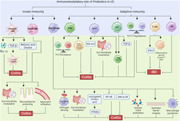

## 1 INTRODUCTION

Ulcerative colitis (UC) is a form of inflammatory bowel disease (IBD) that is experiencing an increasing prevalence on a global scale, with a particular emphasis on developing nations.^1^ UC is a pathologic disease that may be identified through its clinical manifestations, including diarrhea, abdominal pain, and rectal bleeding. Individuals with UC exhibit inflammation and the presence of ulcers in the colon. The exact cause of UC remains uncertain; however, it is believed that an abnormal immune response in the gut contributes to its development.^2^, ^3^ The precise mechanisms underlying the pathology of UC remain unknown. However, a hyperactive immune response to gut bacteria likely plays a significant role in genetically susceptible individuals.^4^ The development of the immune system is intricately connected to the gut microbiota and has been found to be associated with the pathophysiology of UC. Studies have shown that UC patients exhibit lower microbial diversity and dysbiosis.^5^ Furthermore, environmental factors substantially impact the overall development of UC.^5^ While the specific processes involved in the pathophysiology of UC are still unknown, it is recognized that in genetically predisposed people, the condition is related to an increased immune reaction to environmental stimuli or the local gut flora. Given the compromised barrier function and increased permeability of the intestines in UC, maintaining a healthy immune system is crucial.^6^

According to research, immunological dysfunction is a crucial aspect of the pathogenesis of UC.^7^, ^8^, ^9^ Various types of innate immune cells, including neutrophils ^10^ and DCs,^11^ are key factors in the pathological progression of IBD. The decline in epithelial barrier performance and the generation of inflammatory compounds are both characteristics of neutrophils, contributing to gastrointestinal inflammation.^10^ By modulating communication between the innate and adaptive immune systems, DCs serve a critical role in maintaining immunological homeostasis.^11^ Intestinal epithelial cells (IEC) can produce transforming growth factor (TGF)‐β, retinoic acid, and thymic stromal lymphopoietin. These molecules perform an essential function in modulating the activity of DCs and facilitating the generation of interleukin‐10 (IL‐10)‐producing DCs. These IL‐10‐producing DCs elicit immune reactions characterized by anti‐inflammatory properties and the promotion of tolerance.^12^, ^13^

**Figure 1:**
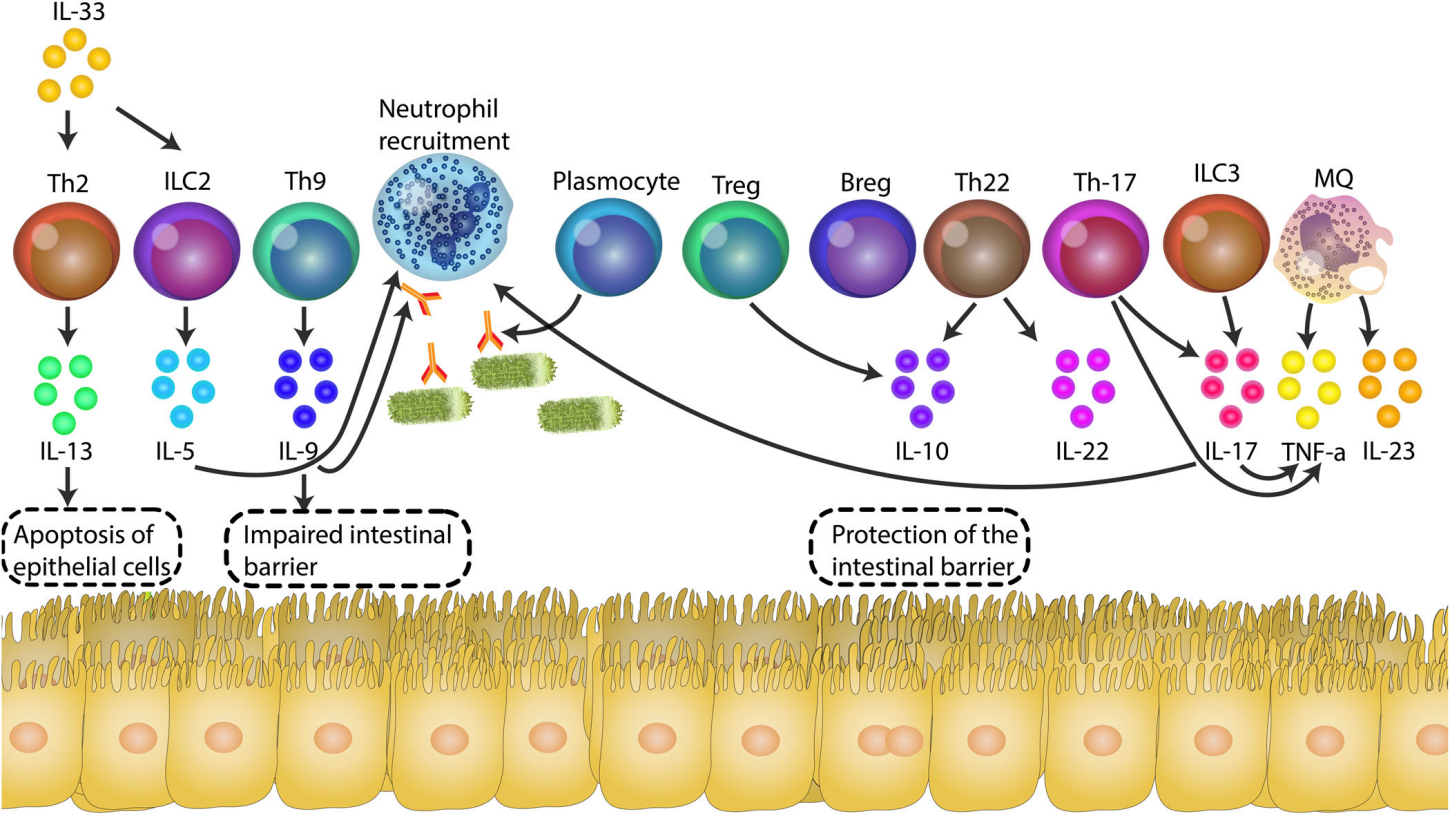
The involvement of immune cells in developing ulcerative colitis (UC) is highly significant. UC patients exhibit an elevated presence of Th2, Th9, and Th17 cells, along with ILC2 and ILC3. These cells release cytokines that contribute to the breakdown of the intestinal barrier by increasing the levels of claudin‐2 and inducing epithelial cell death. Furthermore, these cytokines facilitate the migration and degranulation of neutrophils while also stimulating other immune cells. Activated plasma cells generate antibodies, including those targeting chemokines and IL‐1 produced by the gut microbiota. Unfortunately, the numbers and/or activity of Breg, Treg, and Th22 cells are declining, compromising their ability to safeguard the intestinal barrier effectively.

**Figure 2:**
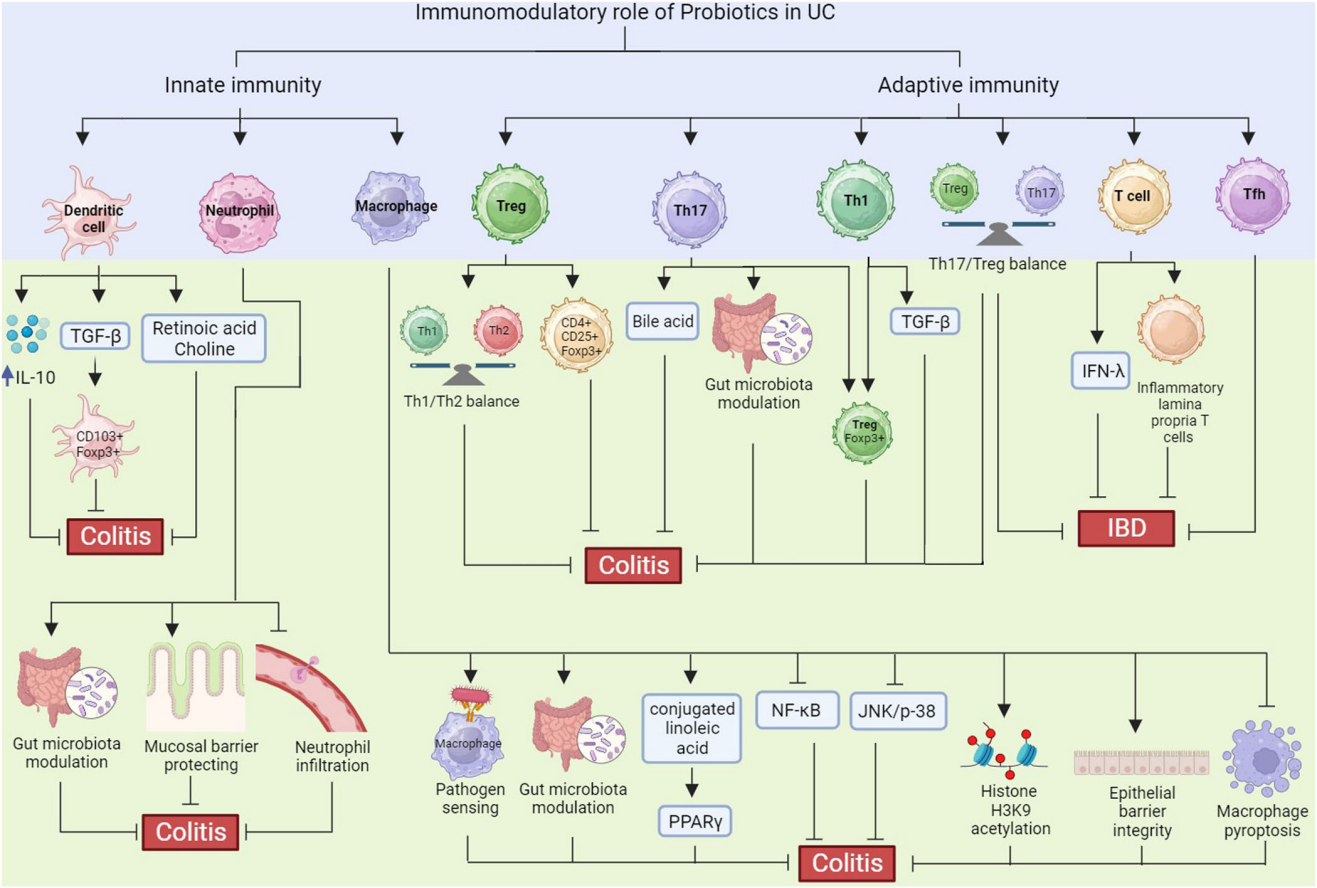
The immunomodulatory role of probiotics on immune cells during ulcerative colitis.

A key target for probiotic treatment in UC has been identified as macrophages, an immune cell type. Macrophages are tissue‐resident phagocytes that control inflammatory processes, protect the host, and preserve tissue homeostasis.^14^ However, macrophages may become dysfunctional in the setting of UC and contribute to the chronic inflammation that characterizes the condition.^14^, ^15^ Macrophages have been associated with IBD pathogenesis in both in vitro investigations using human peripheral monocytes and animal models of intestinal inflammation.^16^, ^17^ Previous research has shown the possible benefits of targeting macrophages therapeutically in IBD. It has been hypothesized that anti‐TNF‐α treatment may reduce the number of macrophages in the intestine,^18^ changing their profile from an inflammatory M1 state to a more restrained M2 state.^19^, ^20^ Recent studies have indicated that probiotics can modulate macrophage function in UC. For instance, VSL#3, a probiotic mixture containing several beneficial bacterial strains, was associated with increased polarization of M2 macrophages and reduced inflammation in a mouse model of UC.^21^ Clinical trials have also demonstrated that VSL#3 improves clinical symptoms and reduces inflammatory markers in UC patients.^22^ These results imply that probiotics have potential as a treatment for UC by modulating macrophage polarization and reducing inflammation. Overall, this review attempts to improve our knowledge of probiotics' therapeutic potential for UC, mainly focusing on their impact on macrophage function. By shedding light on the underlying mechanisms through which probiotics modulate macrophage function in UC, this review might expedite the hunt for effective probiotic treatments for this debilitating disease (Figures 1, 2).

## 2 IMMUNOPATHOGENESIS OF UC

Genetic predisposition, imbalanced immune system responses, compromised mucosal barrier integrity, and environmental variables might involved in the immunopathogenesis of UC.^1^, ^6^, ^10^ This section will provide a detailed discussion of the immunological causes and consequences of colonic inflammation in UC. As mentioned earlier, dysbiosis is believed to be a significant contributing factor to the development of UC, which genetic and environmental variables can influence. Dysbiosis has been linked to immune system activation, disrupted epithelial barrier integrity, and the propagation of inflammatory reactions. Nevertheless, it's crucial to mention immunological dysfunction may be the primary cause of the resulting consequences in UC.^23^ Immune cells, especially those that participate in both adaptive and innate immune reactions, significantly impact the etiology of UC. In the course of the innate immune system's response to UC, macrophages, neutrophils, and DCs are prominently present. Neutrophils, which are more abundant in the blood and colons of patients during active disease phases, are among the primary cell types recruited. These cells contribute to tissue damage, degradation of epithelial barrier function caused by proteolytic and oxidative damage, and the production of inflammatory compounds in UC.^10^ However, DCs and macrophages play a crucial role in antigens' uptake and subsequent processing and presentation to B and T cells.^24^ There is a rise in the generation of cytokines that induces inflammatory processes, such as TNF‐α, IL‐1, IL‐6, IL‐8, IL‐12, IL‐18, and TNF‐like cytokine 1A when macrophages and DCs detect pathogen‐associated molecular patterns.^25^, ^26^ For example, neutrophils are among the initial cells participating in the disease's active phase by releasing neutrophil extracellular traps or degranulating their granules. Neutrophils are the key factors in the inflammatory process in the gastrointestinal tissue in UC. In addition, they produce myeloperoxidase (MPO), cathepsin G, and neutrophil elastase, among other granule components.^27^, ^28^, ^29^

ILCs, located near the intestinal epithelium, contribute to UC. These cells maintain epithelial integrity and defend against infections by producing various cytokines. However, ILCs may struggle to generate anti‐inflammatory cytokines when stimulated by altered microbiota and excessive antigens. This difficulty may lead to the initiation and perpetuation of colon inflammation in UC. In such cases, ILCs may exhibit elevated interferon‐gamma (IFN‐γ) levels, IL‐22, IL‐23, and IL‐17cytokine gene expression and tissue production.^30^, ^31^ Antigen‐presenting cells (APCs) are essential in triggering immune response mechanisms by presenting antigens to B and T cells. Although this immune response takes longer, it is more precise than the innate immune response. Based on the expression of cell surface components CD4 and CD8, lymphocytes are divided into T CD8+ cells, which are mainly cytotoxic, and T CD4+ cells. By producing and releasing inflammation‐promoting cytokines such as TNF‐α and IFN‐γ, the first class of cells contributes to the pathological alterations in UC patients' gut, leading to epithelial cell destruction and intestinal ulcers developing in UC. The latter category includes helper Th and Treg. Th cells are categorized into several subsets, including Th1, Th2, Th9, Th17, and Th22, which are differentiated based on their distinctive cytokine profiles. Particular cytokine environments and the activation of key transcription factors are necessary for naïve cells to differentiate into distinct helper T cell types.^32^, ^33^, ^34^

Previous data suggest that the Th2 response is predominant in UC. GATA binding protein 3 expression is elevated in patients with UC ^35^ and elevated levels of IL‐5‐producing Th2 cells.^36^ Moreover, patient mucosa shows higher concentrations of IL‐10.^37^ However, UC patients' mucosa has decreased cytokine levels known as anti‐inflammatory IL‐4.^38^ These findings indicate that UC may not solely exhibit a disease progression that seems to be influenced by the Th2 response and other cell types. Recent studies also highlight the involvement of Th17, Th22, and Th9 cells and their associated cytokines in UC.^39^, ^40^, ^41^ Cytokines belonging to the IL‐17 family and IL‐21, IL‐22, IL‐12, and TNF‐α are secreted by Th17 cells. The secretion of chemokines (CXCL1, CXCL2, CXCL5, CXCL8) stimulated by IL‐17A attracts neutrophils and lymphocytes to the inflamed tissue. Also, IL‐17A promotes the production of TNF‐α and IL‐1β, two pro‐inflammatory molecules.^33^, ^42^ Genetic polymorphisms in the Th17 cell signaling system components suggest their involvement in the IBD pathophysiology. Investigations on humans have shown that Th17 cells increased cytokine production, including IL‐17A and IL‐17F, in the colons of IBD patients. In conjunction with IL‐21, the cytokines mentioned above play a pivotal role in recruiting neutrophils and enhancing damage to the intestinal epithelium. The induction of inflammation, which contributes to intestinal epithelium injury, is facilitated by cytokines such as TNF‐α, IL‐1, IL‐8, and IL‐6.^43^, ^44^

Additionally, IL‐22 supports the functioning of the intestinal barrier by promoting mucus and antimicrobial peptide production.^33^, ^45^ Human Th17 cells possess a dual purpose in the body: they release cytokines that promote inflammation (such as IL‐17, TNF‐α) while also secreting protective mediators for the intestinal epithelium (such as IL‐10, IL‐22). With increased stimulation, Th17 cells can become pathogenic, leading to persistent inflammation and the onset of UC.^42^, ^46^ In brief, UC can arise due to an amplified immune response characterized by the involvement of macrophages, Th2, Th9, Th17 cells, neutrophils, and pro‐inflammatory cytokines. Additionally, alterations in the gut microbiota and impairments in mucus and epithelial barrier function may contribute to the development of UC. Understanding these immunological factors is crucial for developing targeted therapies to modulate immune responses, restore barrier function, and maintain mucosal homeostasis in UC patients. More study is required to determine UC immunopathogenesis's intricate mechanisms and identify novel therapeutic targets.

## 3 MECHANISMS OF IMMUNE MODULATION BY PROBIOTICS

Researchers have looked at the effectiveness of probiotics in reestablishing gut microbial balance, which is essential for immunological homeostasis.^47^, ^48^, ^49^ Recent studies have highlighted the possibility that probiotics or their metabolites might communicate with various types of immune cells, bestowing onto them the ability to regulate the immune system.^50^, ^51^, ^52^ Probiotics, for instance, aid in developing the humoral immune system. Probiotics that enter the gut may increase IgA antibody synthesis.^53^ Probiotics have also been demonstrated to boost lamina propria‐associated immune cells and the innate response and cytokines generated by T cells. The results show probiotics might boost immunological function.^54^, ^55^ The impact of probiotic immunomodulation on innate and adaptive immune cells during UC is shown in Table 1.

**Table 1:** The effect of probiotic immunomodulation on innate and adaptive immune cells during UC.

UC	Study setting	Probiotic	Target immune cell	Conclusion	References
	DSS‐induced colitis model	VSL#3	Macrophage	VSL#3 produces conjugated linoleic acid locally in the gut that targets macrophage PPARγ to suppress colitis	[56]
	Human monocyte‐derived macrophages	VSL#3	Macrophage	VSL#3 induces mixed pro‐inflammatory and anti‐inflammatory phenotypes in polarized and unpolarized macrophages	[57]
	DSS‐induced colitis model	*Clostridium butyricum*‐derived EVs	Macrophage	*C. butyricum*‐derived EVs could protect against DSS‐induced colitis by regulating the repolarization of M2 macrophages and remodeling the composition of gut microbiota	[58]
	TNBS	*Leuconostoc lactis EJ‐1*	Macrophage	*EJ‐1* inhibits the NF‐κB signaling and polarizes M1‐ to M2‐macrophage transition, which helps in alleviating colitis	[59]
	TNBS	VSL#3	Macrophage	VSL#3 reduces colitis severity, colonic macrophage infiltration, and serum cytokine levels but does not dampen the pro‐inflammatory phenotype of M1 macrophages	[21]
	RAW264·7 macrophage cell line	*Escherichia coli (EC) strain M‐17*	Macrophage	*EC‐M17* improves murine colitis, probably due to an inhibitory effect on NF‐κB signaling	[60]
	DSS‐induced colitis model	*Saccharomyces cerevisiae*	Macrophage	*S. cerevisiae* attenuated DSS‐induced colitis in mice by suppressing macrophage pyroptosis and modulating the intestinal microbiota, an effective and safe treatment strategy for ulcerative colitis	[61]
	Caco‐2 epithelial cell line	*Lacticaseibacillus casei Strain*	Macrophage	*Lacticaseibacillus casei Strain* selectively modulates epithelial barrier integrity, pathogen sensing, and inflammatory cytokine profile, determined by macrophage subset and activation status	[62]
	RAW264.7 cells and DSS‐induced colitis model	*Lactobacillus casei LH23*	Macrophage	*L. casei LH23* regulates the immune response and improves DSS‐induced colitis by inhibiting JNK/p‐38 signal pathways and increasing histone H3K9 acetylation	[63]
	DSS‐induced colitis model	*Zygosaccharomyces sapae strain* I‐6	DC	Strain I‐6 caused phenotypic alterations in intestinal CD11c+ DCs, including increased IL‐10 production, and had substantial anti‐inflammatory impacts on DSS‐induced colitis	[64]
	DSS‐induced colitis model	*Lactiplantibacillus plantarum 22A‐3*	DC	*L. plantarum 22A‐3* stimulates intestinal epithelial cells, leading to TGF‐β1 production and activation of CD103+ and Foxp3+ dendritic cells—Development of Tregs and their potential to improve murine colitis	[65]
	DSS‐induced colitis model		DC	*Enterobacter ludwigii* has been shown to enhance the immunotolerance of DCs to form Treg through choline and retinoic acid‐mediated a7nAChR and upregulation of TGF and protect mice against DSS‐induced colitis	[66]
	DSS‐induced colitis model	VSL#3	T cells	VSL#3 alleviates DSS‐induced colitis by downregulating Tfh cells, and Tfh cells may become a potential therapeutic target for IBD	[67]
	TNBS	Bifico capsules (combined *bifidobacterium, lactobacillus*, and *enterococcus*)	Tregs	Bifico capsules effectively treat experimental colitis by increasing CD4+CD25+Foxp3+T cells and regulating the balance of Th1 and Th2 cytokines in colonic mucosa	[68]
	TNBS	*Bifidobacterium bifidum FJSWX19M5*	Tregs	The symptoms of chronic colitis induced by TNBS were alleviated by the modulation of gut barrier integrity and the enhancement of regulatory T cells (Tregs) by *B. bifidum FJSWX19M5*	[69]
	DSS‐induced colitis model	*Lactobacillus paracasei R3*	Th17/Treg	Through modulating the Th17/Treg cell balance in DSS‐induced colitis in mice, *L. paracasei R3* considerably alleviates colitis symptoms and pathological impairment	[70]
	DSS‐induced colitis model	*Lactobacillus acidophilus*	Th17/Treg	*L. acidophilus* might be a potential therapy for IBD by altering the balance of Th17 and Treg cells and the progression of fibrosis	[71]
	DSS‐induced colitis model	*Porphyromonas gingivalis* and *Lactobacillus rhamnosus* GG	Th17/Treg	Through TLR4 and TLR2, *P. gingivalis* and *L. rhamnosus* GG modulate colitis's Th17/Treg balance	[72]
	TNBS	*Lactobacillus plantarum* C29	Th17/Treg	*L. plantarum C29* has been shown to possess the ability to mitigate colitis via the modulation of NF‐κB activation and the balance between Th17 and Treg immune cell populations	[73]
	DSS‐induced colitis model	*Streptococcus thermophilus ST28*	Th17	*ST28* inhibits the Th17 response in inflamed intestines and might be used for alleviating Th17‐mediated disorders like IBD	[74]
	DSS‐induced colitis model	*Bacteroides uniformis*	Th17	The alterations in colonic microbiota and bile acid levels generated by *B. uniformis* have been shown to impede the differentiation of Th17 cells and improve the progression of colitis	[75]
	TNBS	*Bifidobacterium infantis*	Th1 and Th17	The administration of *B. infantis* has been shown to successfully mitigate colitis produced by TNBS by reducing the activation of Th1 and Th17 immune responses and promoting the expansion of Foxp3+ Treg cells in the colon's mucosal layer	[76]
	Mucosal biopsies and surgical samples	*Lactobacillus kefiri*	T cells	*L. kefir* reduces inflammatory lamina propria T cells from patients with active IBD	[77]
	Caco‐2/Jurkat T cell coculture model and a scaffold‐based 3D coculture IBD model	*E. coli Nissle 1917*	T cells	This study showed the anti‐inflammatory effects of interferon lambda 1‐expressing probiotics in two in‐vitro IBD models, demonstrating their potential as live biotherapeutics for IBD immunotherapy	[78]
	DSS‐induced colitis model	*L. plantarum CBT LP3*	T cells	*L. plantarum CBT LP3* can be used as a potent immunomodulator, which has significant implications for IBD treatment	[79]
	TNBS	VSL#3	Th1	VSL#3 administration during a remission period ameliorates the severity of recurrent colitis by inducing an immunoregulatory response involving TGF‐beta‐bearing regulatory cells	[80]
	DSS‐induced colitis model	Synbiotics	Neutrophil	The administration of synbiotics in experimental UC leads to a decrease in the infiltration of mucosal inflammatory neutrophils and an increase in hematocrit	[81]
	DSS‐induced colitis model	*L. plantarum HNU082* (*Lp082*)	Neutrophil	This study shows that *Lp082* has a therapeutic effect on colitis in mice. Its mechanisms of action include protecting the mucosal barrier and actively modulating the intestinal microbiome, modulating inflammatory pathways, and reducing neutrophil infiltration.	[82]

Abbreviations: DSS, dextran sulphate sodium; UC, ulcerative colitis.

### 3.1 Innate immunity

The innate immune system acts as the body's initial defense against infections, involving various components such as chemical barriers, the microbiota, mucous membranes, skin, and specialized cells promoting foreign pathogens' engulfment.^83^, ^84^ Several *Bifidobacterium probiotic* strains, including *Bifidobacterium adolescentis*, *B. infantis*, *Bifidobacterium longum*, and *Bifidobacterium bifidum*, can potentially regulate cell death in IEC. Moreover, they can increase mucin secretion, which acts as the primary protective barrier against infectious agents in the intestines.^85^ DCs and epithelial cells are particularly significant in probiotic research, as they are the first to encounter harmful compounds produced by the gut microbiota. DCs found in the gastrointestinal tract's mucosa and in the gut‐associated lymphoid tissue (GALT) are called “detector cells” due to their specific receptors that bind to certain sites on pathogens. These cells also catalyze signaling pathways, such as c‐type lectin receptors and Toll‐like receptors (TLRs), that modify their characteristics and release cytokines.^86^ For instance, the probiotic strain *B. infantis* 35624 can influence dendritic cell function by increasing the population of cDC1 (CD103+DC) in the intestinal epithelial layer. Human studies have shown that this strain has several positive effects on health, including lowering the incidence of colitis caused by dextran sulphate sodium (DSS) through a retinoic acid‐dependent mechanism.^87^ Additionally, *Lactobacillus rhamnosus* JB‐1 inhibits the expression of co‐stimulatory molecules, cytokine production and maturation, and the differentiation of Th1/Th17 cells by stimulating human monocyte‐derived dendritic cells. This particular strain, which expresses Foxp3 and induces the production of IL‐10, possesses immunomodulatory properties.^88^ The cell wall components of probiotic bacterial strains also play a role in the immunomodulation of dendritic cells. The generation of IL‐10 from T helper cells is stimulated when the capsular polysaccharide binds to TLR‐2 receptors on DCs. This reduces the inflammation that occurs in colitis.^88^ Furthermore, *Lactobacillus casei* and *L. rhamnosus* prevent enterocytes from producing cytokines that promote inflammation in response to *Clostridium difficile* infection, while the infection‐induced production of natural killer cells is stimulated by *Lactobacillus plantarum*, *L. casei*, *Lactobacillus paracasei* ssp. *paracasei*, *B. lactus*, *B. animalis* ssp. lactis, and *B. polyfermenticus*.^89^ In conclusion, probiotics have shown the ability to modify a variety of immune system functions, especially those that relate to innate immunity. They interact with specialized cells, such as dendritic cells and epithelial cells, promoting immune signaling pathways and influencing the production of cytokines. Moreover, probiotic strains, including *Bifidobacterium* and *Lactobacillus* species, have shown potential in enhancing mucin secretion, regulating apoptosis in IEC, and reducing inflammation, thereby contributing to the maintenance of a healthy immune response.

### 3.2 Adaptive immunity

T cells and B cells are examples of adaptive immune system cells that have been shown to interact with probiotics, which can lead to changes in their function and response.^90^, ^91^ Probiotic bacteria alter the gut microbiota, which helps to increase the activity of immune cells like Treg cells, B cells, Th17, Th2, and Th1. This helps to manage immunological‐mediated diseases that are unique to the emergence of diseases.^91^ In terms of B cells, oral administration of *Bifidobacterium breve* increased the exposure of IgA to cholera toxin, while *B. bifidum* enhanced the humoral immune response to egg albumin.^92^ The potential of probiotics to strengthen the population of Treg cells is a contributing factor to their anti‐inflammatory and anti‐colitis activities. For example, *B. longum* is effective in the treatment of colorectal colitis in mice by promoting the production of T‐regulated lymphocytic cells, which results in a decline in cytokines associated with inflammation like IL‐23, IL‐12, and IL‐27 while an increase in IL‐10 and IL‐12 levels in the bloodstream.^93^ Probiotic bacteria produce a collective of compounds called short‐chain fatty acids (SCFAs), including butyrate, acetate, and propionate. These SCFAs have a role in regulating T‐cell homeostasis, either via direct or indirect mechanisms. Propionate inhibits T‐cell growth by blocking histone deacetylase, while butyrate increases the formation of forkhead box P3 (FOXP3)+cells and Treg cells outside of the hypothalamus. The increase in the number of Th17 inflammatory cell types, which have been related to the emergence and advancement of inflammatory conditions such as irritable bowel syndrome, has been demonstrated to be inhibited by probiotics like *B. longum, Lactobacillus gasseri*, *B. breve*, *Lactobacillus acidophilus*, and *B. longum subsp. infantis*.^94^ Furthermore, it has been shown that *L. rhamnosus GG* and *B. breve* can potentially decrease the activity of IL‐17 and IL‐23. These two crucial factors play a vital role in the formation, viability, and activation of Th17 cells. Different *Lactobacillus* and *Bifidobacterium* species produce TNF‐α and IFN‐γ, and they stop Th17 inflammatory cells from proliferating. Increased IL‐27 synthesis by *B. longum* has been connected to fewer Th17 cells that can produce IL‐17.^95^ Probiotics may change the immune system's reaction from a Th2 to a Th1 one. *L. casei* may increase IL‐12 creation, which encourages a Th1 response and reduces the severity of Th2‐related illnesses. Seasonal allergies, atopic dermatitis, and psoriatic arthritis all have clinical symptoms that are improved by *L. rhamnosus*, which also lowers Th2 and Th17 cells.^96^ In conclusion, probiotics have been shown to interact with and influence the function of components of adaptive immunity, including B cells and T cells. Probiotics may play a part in controlling immune response problems related to the etiology of diseases by altering the gut microbiota and activating immune cells, including B cells, Treg cells, Th17, Th2, and Th1. The number of T‐regulated lymphocytic cells can be increased by probiotics, and they can also make SCFAs that control T‐cell homeostasis, stop the growth of Th17 inflammatory cells, and change the immune response from Th2 to Th1 to generate anti‐inflammatory and immunomodulatory outcomes. To fully understand the therapeutic potential of probiotics in conditions such as UC and other immune‐related disorders, it is essential to grasp the intricate mechanisms through which probiotics influence immune cell modulation.

## 4 EFFECTS OF PROBIOTICS ON MACROPHAGES IN UC

Macrophages are essential in both the onset and development of UC.^8^, ^14^, ^97^, ^98^ In UC, macrophages are implicated in initiating and sustaining inflammatory reactions. They migrate to inflamed colon tissues and release pro‐inflammatory molecules, including reactive oxygen species (ROS), TNF‐α, IL‐6, IL‐1β, and nitric oxide (NO). These molecules damage tissue and perpetuate inflammation (3‐4). Increasing evidence suggests that probiotics might modify the activity and function of macrophages in UC.^58^, ^59^, ^61^ Fitzpatrick and colleagues undertook a study to evaluate the impact of a probiotic strain of *E. coli* known as *M‐17* (*EC‐M17*) on the immune response and the occurrence of colitis in mice.^60^ Scientists investigated the impact of *EC‐M17* on NF‐κB signaling, cytokine production, and its efficacy in a DSS‐induced mice model of colitis.^60^ NF‐κB signaling, a critical pathway regulating the immune response, was assessed using a luciferase reporter cell line provoked with TNF‐α. The interaction between the protein p65 and the nucleus and the subsequent synthesis of cytokines that promote inflammation, including TNF‐α IL‐6, and IL‐1β, were evaluated in a macrophage cell line exposed to LPS.^60^ The study involved administering *EC‐M17*, metronidazole (an antibiotic commonly used to treat colitis), or a combination of both to mice with DSS‐induced colitis for 13 days.^60^ The effectiveness of the treatments was evaluated by assessing the DAI, MPO activity, histology, and NF‐κB p65 levels. The results demonstrated that EC‐M17 dose‐dependently inhibited NF‐κB signaling induced by TNF‐α, with an inhibition rate of over 95% observed at a concentration of 5 × 10^9 colony‐forming units/ml. In the macrophage cell line, *EC‐M17* significantly inhibited LPS‐induced nuclear p65 binding by 78% at a concentration of 1 × 10^8 colony‐forming units/mL.^60^ Furthermore, the production of IL‐6, IL‐1β, and TNF‐α was substantially lowered by *EC‐M17* in the macrophage cell line, with inhibition rates exceeding 90%. In the murine model of colitis induced by DSS, both *EC‐M17* and metronidazole demonstrated individual efficacy in reducing the disease activity index (DAI) and improving colonic histology scores.^60^ Colonic cytokines that promote inflammation, including IL‐1β, IL‐12, IFN‐γ, and IL‐6, were also reduced by this therapy. Notably, when *EC‐M17* and metronidazole were used together, the effects on cytokine levels were more evident than when each medication was used alone.^60^ Furthermore, combining both treatments considerably decreased colonic histology scores compared to metronidazole alone and lowered concentrations of IL‐1β compared to *EC‐M17* alone. Overall, the study demonstrated that *EC‐M17* exhibits immunomodulatory effects by inhibiting NF‐κB signaling and suppressing the secretion of pro‐inflammatory cytokines. Additionally, *EC‐M17* has shown effectiveness in reducing colitis in mice, whether used alone or in conjunction with metronidazole. The results imply that the medicinal value of *EC‐M17* in the amelioration of colitis is influenced by its inhibitory impact on NF‐κB signaling.^60^

Bassaganya‐Riera and colleagues conducted research to understand the mechanisms through which probiotic bacteria exert anti‐inflammatory benefits in a mice model of colitis.^56^ This study is significant as it attempts to shed light on cellular and molecular processes underpinning the possible benefits of probiotics for treating UC. The researchers performed experiments using VSL#3 and conjugated linoleic acid (CLA) in a DSS‐induced mice model of colitis.^56^ They collected colonic tissue samples for various analyses, including flow cytometry, histopathology, and gene expression. They also looked at immune cell subsets in several tissues, including the blood, colonic lamina propria cells, mesenteric lymph nodes, and spleen, to better comprehend how the probiotic bacteria and CLA influenced the immune system. To evaluate the effects of VSL#3 and CLA on gut bacteria diversity and CLA generation, colonic contents and stool specimens were also examined.^56^ The study's findings revealed that both VSL#3 and CLA treatments improved colitis symptoms and reduced colonic bacterial diversity.^56^ The injection of probiotic bacteria was shown to be correlated with the increased presence of CLA in the colon, although it did not spread systemically to the bloodstream. Furthermore, CLA and VSL#3 treatments reduced the aggregation of macrophages in the mesenteric lymph nodes of mice with DSS‐induced colitis. Notably, the protective effects of probiotic bacteria and CLA were abolished when the myeloid cells lacked PPAR γ.^56^ Based on these findings, the study concludes that probiotic bacteria modulate gut microbiota diversity and promote CLA's localized production in the colon. This localized production of CLA specifically targets PPAR γ in myeloid cells, suppressing colitis.^56^ The significance of this study lies in advancing our understanding of the therapeutic mechanisms of probiotics in the context of colitis. It provides valuable insights into how probiotic bacteria and their metabolites can modulate gut microbial diversity, exert anti‐inflammatory effects, and specifically target immune cells involved in colitis pathology. This understanding brings to the creation of possible therapeutic options for treating colitis and other IBD.

Abdelouhab and colleagues conducted a recent study focusing on investigating the role of NO production in the pathophysiology of IBD, specifically UC.^99^ This study is significant as it examines the possible therapeutic advantages of prebiotics and probiotics in attenuating immune disorders associated with IBD and their impact on NO production and colonic mucosa. The researchers induced acute UC in Swiss mice by administering 3% DSS and evaluated the preventive effects of two probiotics, inulin and Ultrabiotique®, on colitis. They measured NO output in the culture supernatants of macrophages from the peritoneum (pMφ), which plays a role in the immunological response.^99^ The severity of colitis was also evaluated through an examination of the colonic mucosa's histology. The study's findings revealed that the administration of DSS led to severe acute UC in the mice and significant increases in NO generation in pM cultures higher than in control samples. This finding was linked to severe mucosal damage in the colon, suggesting a potential role for NO in the development of experimental UC. In contrast, oral administration of Ultrabiotique® or inulin reduced the severity of DSS‐induced colitis. In pMφ cultures, these procedures led to a decline in NO and a substantial decrease in colonic lesions. As a result, the research reveals that probiotics and prebiotics have an anti‐inflammatory impact that is probably mediated by a change in NO generation.^99^ This research hinges on providing insights into the function NO in the emergence of experimental UC and its correlation with mucosal intestinal alterations. It highlights the potential of probiotics and prebiotics as therapeutic strategies for attenuating immune disorders associated with IBD. By demonstrating the ability of these interventions to decrease NO production and alleviate colitis symptoms, the study contributes to the comprehension of the principles underpinning the protective benefits of prebiotics and probiotics in IBD treatment. This understanding might influence the creation of innovative therapeutic strategies that aim to control NO generation for treating UC and other IBD.^99^

An investigation by Isidro and colleagues explored the impact of the probiotic VSL#3 on the phenotype of human macrophages, which significantly influence IBD, such as UC and CD.^57^ The significance of this study lies in its ability to offer illuminating viewpoints on the effects of VSL#3 on the polarization and secretion profile of human macrophages. This leads to an improved comprehension of the fundamental principles that underlie probiotics' therapeutic potential in IBD.^57^ The study involved the generation of human monocyte‐derived macrophages, which were subsequently polarized towards three distinct phenotypes: M1 pro‐inflammatory, M2 anti‐inflammatory, and unpolarized MΦ. These macrophages were cultured with or without VSL#3 for 3 days, and macrophage morphology and secretion profile changes were assessed. Supernatants from cultures of macrophages were analyzed for their concentrations of cytokines and chemokines.^57^ The study's findings showed that VSL#3 has a variety of impacts on the phenotypic and secretory profile of macrophages. VSL#3 enhanced the presence of fibroblast‐like M2 macrophages, diminished the fibroblast‐like phenotype of unpolarized M macrophages, and lessened the development of granuloma‐like aggregates in M1 macrophages.^57^

Additionally, VSL#3 altered how macrophages secreted cytokines and chemokines. It caused M1, M2, and M macrophages to secrete more pro‐inflammatory cytokines such as G‐CSF, IL‐6, IL‐1β, and IL‐10. The M1 macrophages were maintained by VSL#3 administration by maintaining IL‐12 production, boosting IL‐23 secretion, and reducing macrophage‐derived chemokine secretion. In contrast, M2 and M macrophages that were subjected to VSL#3 treatment exhibited a significant increase in the production of anti‐inflammatory, pro‐healing, and pro‐inflammatory cytokines, respectively.^57^ The investigation suggests that VSL#3 induced a combination of pro‐inflammatory and anti‐inflammatory characteristics in both polarized and unpolarized human macrophages. This divergent outcome might explain why probiotic treatment may not be as effective for individuals with CD, who have elevated M1 macrophages, as it is for those with UC, who have a predominance of Th2 cytokines.^57^ These results illuminate possible mechanisms by which probiotics exert their therapeutic benefits in IBD and provide significant new insights into the complicated relationships between probiotics and human macrophages.^57^ The study's importance rests in enhancing our knowledge of the effects of VSL#3 on macrophages, which are key immune cells implicated in IBD pathophysiology. The findings contribute to our understanding of how probiotics can regulate immunological responses in the context of IBD, providing a rationale for the differential response of CD and UC patients to probiotic therapy. This knowledge may guide the development of more targeted and effective probiotic interventions for the management of IBD.

Isidro and colleagues looked at the processes behind the therapeutic benefits of the probiotic mixture VSL#3 in acute colitis induced by 2,4,6‐Trinitrobenzene sulfonic acid (TNBS).^21^ This study is significant as it aims to understand how VSL#3 influences macrophages, inflammation, and gut microbiota in the context of colitis. The researchers conducted experiments using rats randomized into three groups: normal, colitis alone, and colitis with VSL#3 treatment.^21^ The levels of cytokines in the serum were evaluated, and the rats' colons were examined for macroscopic and microscopic damage. Analyses of the microbiome were also conducted. The research also looked at the transcript levels of barrier, anti‐barrier, and pro‐inflammatory proteins in the colon.^21^ The investigation findings showed that rats given VSL#3 alongside TNBS‐induced colitis exhibited reduced macroscopic and microscopic damage in the proximal colon compared to rats with colitis alone.^21^ Increased macrophage infiltration into the colon due to colitis was reversed by VSL#3 treatment.^21^ Although the administration of VSL#3 did not mitigate the pro‐inflammatory phenotype of hepatic or colonic M1 macrophages, it ultimately decreased the total number of these cells. The VSL#3 administration resulted in a reduction in blood concentrations of cytokines, as well as the restoration of colonic transcript levels for several barrier proteins, anti‐inflammatory and inflammatory, putting them to either high or normal levels. Additionally, there were differences in the groups' fecal microbial distributions.^21^ In conclusion, the research shows that VSL#3 lessens the severity of colitis, serum cytokine concentrations, and colonic macrophage infiltration. However, the pro‐inflammatory characteristics of M1 macrophages remain intact. Additionally, VSL#3 results in the restoration of colonic transcript levels for significant proteins involved in controlling inflammation and the operation of the gut barrier. The findings illuminate the methods through which VSL#3 exerts its anti‐inflammatory and anticolitis benefits, emphasizing its capacity to control macrophages, inflammation, and gut microbiota.^21^ The significance of this study lies in expanding our understanding of the principles that underlie the pharmacological potential of VSL#3 in colitis. By elucidating its effects on macrophages, inflammation, and the gut microbiota, the study provides important insights into the complex interactions between probiotics and the immune system in the context of colitis. This knowledge contributes to the advancement of targeted and efficient probiotic interventions for the management of IBD.

Jang and colleagues researched to understand the potential therapeutic effects of *Leuconostoc lactis EJ‐1* (*L. lactis EJ‐1*), a specific strain of *lactic acid bacteria* (*LAB*) found in kimchi, on colitis, a chronic inflammatory disease of the intestines.^21^ The study aimed to determine a nutritional element that could inhibit the inflammatory signaling pathway called NF‐κB and alleviate symptoms of IBD.^21^ The researchers in peritoneal macrophages stimulated by LPS first examined the ability of *EJ‐1* to suppress the production of IL‐1β, TNF‐α, and IL‐6.^21^ They then evaluated the potential therapeutic effects of the *EJ‐1* TNBS mice model of colitis. The colitis model exhibited increased inflammation and tissue damage, as indicated by elevated levels of MPO, colon shortening, and macroscopic colitis scores. However, *L. lactis EJ‐1* was administered orally and caused various beneficial outcomes.^21^ The administration of *EJ‐1* prevented body weight loss, colon shortening, and MPO activity, suggesting a reduction in inflammation and tissue damage.

Moreover, *EJ‐1* hampered the expression of NF‐κB and iNOS and reduced cyclooxygenase‐2 (COX2) levels, further confirming its anti‐inflammatory properties.^21^ The effect of *EJ‐1* on the production of TNF‐α, IL‐1 β, IL‐6, and IL‐10 was also investigated during the study. EJ‐1 administration led to decreased TNF‐α, IL‐6, and IL‐1β levels, which are typically associated with inflammation, while it stimulated the expression of IL‐10, which possesses anti‐inflammatory properties.^21^ Notably, the study observed a shift in macrophage polarization from M1 to M2. *EJ‐1* treatment increased the expression of markers associated with M2 macrophages, including arginase I, IL‐10, and CD206, suggesting a transition toward M2 macrophages.^21^ In summary, this study demonstrates that *L. lactis EJ‐1*, a strain of LAB found in kimchi, possesses anti‐inflammatory properties and can ameliorate mouse colitis.^21^ The beneficial effects of *EJ‐1* appear to be mediated through the suppression of NF‐κB signaling, reduction of pro‐inflammatory cytokines, and promotion of M1‐to‐M2 macrophage polarization. These findings provide valuable insights into potential dietary interventions for managing and treating IBD.

In a recent study, Sun and colleagues researched the lactic acid biosynthesis pathway in *Saccharomyces cerevisiae* and its potential therapeutic effects in treating UC.^61^ The researchers successfully reconstructed a pathway in *S. cerevisiae* that allowed for the direct production of lactic acid from glucose, achieving high lactic acid production. They then evaluated the engineered *S. cerevisiae* strain for its anti‐inflammatory activity using a mouse model of UC induced by DSS.^61^ The study demonstrated that the engineered *S. cerevisiae* strain had beneficial effects in the colitis model. It led to improvements in histological damage, enhanced the integrity of the mucosal barrier, and decreased the intestinal immune response, indicating its potential to alleviate the symptoms of UC.^61^ The lactic acid produced by the engineered *S. cerevisiae* strain was found to play a role in regulating the polarization state of macrophages, a type of immune cell, and suppressed the production of cytokines that contribute to the inflammatory response, both in vivo and in vitro. This suggests that lactic acid possesses anti‐inflammatory properties and can modulate immune responses in colitis.^61^

The study further revealed that increasing lactic acid uptake by macrophages through the macrophage monocarboxylic acid transporter prevented macrophages from inappropriately activating the NLRP3 inflammasome and its downstream caspase‐1 pathway. This indicates that lactic acid can mitigate macrophage‐mediated inflammation by inhibiting specific signaling pathways involved in the inflammatory response.^61^ Additionally, lactic acid was found to promote specific modifications of histone proteins, namely histone H3K18 acetylation and histone H3K9 acetylation. These modifications can potentially influence gene expression and contribute to the observed anti‐inflammatory effects.^61^ Along with its immediate effects on the immune system, the engineered *S. cerevisiae* strain was found to impact the diversity and composition of the intestinal microbiota in mice with colitis. It also altered the abundance of metabolic products produced by the microbiota. This suggests that the therapeutic effects of the strain may involve modulating the gut microbiota, which can influence inflammation and overall intestinal health.^61^ In summary, the results of this research show that using a genetically modified strain of *S. cerevisiae*, capable of directly producing lactic acid, can attenuate colitis in a mouse model. The beneficial effects are attributed to the suppression of macrophage‐mediated inflammation, modulation of the intestinal microbiota, and the anti‐inflammatory properties of lactic acid. These findings highlight the potential of using engineered *S. cerevisiae* as an effective and safe treatment strategy for UC.^61^

In a recent study, Foey and colleagues intended to look into the influence of *L. casei strain Shirota* (LcS) on the immune response, cytokine production, pattern recognition receptor (PRR) expression, and barrier integrity in a co‐culture system.^62^ Macrophages are essential for the immune system's reaction and can exhibit distinct functional states based on their subset polarization and encountered stimuli. Additionally, the interaction between macrophages and IEC is crucial for maintaining the integrity of the gut barrier. This study aimed to explore how *LcS* affects these interactions and subsequent immune responses.^62^ Using a co‐culture system consisting of M1 and M2 subset macrophages derived from THP‐1 cells and differentiated Caco‐2 epithelial cells, the researchers assessed cytokine responses, PRR expression, and barrier integrity in the presence or absence of enteropathogenic LPS, a bacterial component known to induce inflammation.^62^ The study revealed that Caco‐2 cells in monoculture expressed distinct cytokine profiles, PRRs, and barrier integrity, which were influenced by the inflammatory context. LcS exhibited differential effects on the co‐culture system depending on the macrophage subset and activation status. Specifically, *LcS* restored the integrity of the tight junction protein ZO‐1 and the trans‐epithelial electrical resistance (TEER) in the M2/Caco‐2 co‐culture but not in the M1/Caco‐2 co‐culture.^62^ Moreover, *LcS* modulated the expression of PRRs, such as TLR2, TLR4, and myeloid differentiation factor 2, in both co‐cultures, indicating its influence on pathogen sensing. Additionally, LcS regulated the expression of nucleotide‐binding oligomerization domain containing 2, TLR9, Toll‐interacting protein, and cytokine secretion differentially.^62^ In conclusion, this study demonstrates that *LcS* selectively modulates the integrity of the epithelial barrier, the sensing of pathogens, and the inflammatory cytokine profile in a co‐culture system. These effects were dependent on the subset and activation status of macrophages. The findings contribute to understanding the interactions between probiotic bacteria, immune cells, and the intestinal epithelium. They suggest that *LcS* has the potential to influence immune responses and barrier function in the gut, highlighting its significance as a probiotic strain for maintaining intestinal health.^62^

A study by Liu and colleagues examined the potential therapeutic benefits of using *L. casei LH23* (*L. casei LH23*) as a probiotic in mice colitis caused by DSS.^63^ The research study observed that the strain *L. casei LH23* exhibited the ability to suppress the generation of NO and pro‐inflammatory mediators in RAW264.7 cells when stimulated by lipopolysaccharides (LPS).^63^ The observed reduction of the JNK/p38 signaling pathway, recognized for its involvement in inflammation, was shown to be related to the suppression of inflammatory reactions.^63^ In the context of an in vivo model of colitis produced by DSS, the treatment of *L. casei LH23* demonstrated a considerable amelioration of the mice's condition.^63^ Additionally, the administration of LH23 resulted in a decrease in the population of macrophages (CD11b+F4/80+) in the colon and a reduction in the production of inflammatory mediators by these cells.^63^ A research project conducted by Liu and colleagues demonstrated that the administration of *LH23* resulted in an augmentation of the CD3+CD4+CD25+ regulatory T cell population inside the mesenteric lymph nodes. Tregs are recognized for their capacity to modulate the immune response.^63^ Moreover, the administration of *LH23* therapy increased the concentrations of SCFAs, which have been linked to the promotion of gastrointestinal health and the modulation of immunological responses.^63^ Significantly, they discovered that the administration of DSS significantly reduced the degree of histone H3K9 acetylation within the colon tissues, suggesting a disruption in the epigenetic mechanism.^63^ In summary, A study by Liu and colleagues established that *L. casei LH23* had promising therapeutic properties in preventing and treating IBD. The mechanisms of action include the suppression of inflammatory responses by macrophages through the JNK/p38 pathway, the promotion of regulatory T cells, the elevation of SCFA levels, and the reinstatement of histone H3K9 acetylation. The results of this study indicate that *LH23* has potential immunomodulatory effects and the ability to influence epigenetic variables in the setting of IBD. This study enhances our comprehension of the potential use of probiotics as therapies for inflammatory conditions, such as IBD.

In conclusion, probiotics have emerged as a promising therapeutic approach for UC due to their effects on macrophages, key immune cells playing a role in developing the disease. Probiotics can modulate macrophage polarization, promoting a shift from the M1 phenotype toward the M2 phenotype. This shift is connected to a decrease in inflammation and the promotion of tissue repair in the colon. Additionally, probiotics can influence macrophage cytokine production by inhibiting the release of TNF‐α, IL‐1β, and IL‐6. This modulation of cytokine production helps to dampen the inflammatory response in UC and contributes to the resolution of symptoms. Furthermore, probiotics have been found to regulate the expression of PRRs in macrophages. By modulating PRR expression, probiotics enhance the ability of macrophages to appropriately sense and respond to microbial pathogens, thereby preventing excessive inflammation and maintaining immune homeostasis. The collective effects of probiotics on macrophages in UC, including their impact on polarization, cytokine production, and PRR expression, contribute to attenuating inflammation, restoring gut homeostasis, and improving UC symptoms. While mounting evidence supports the favorable impacts of probiotics on macrophages in UC, more investigation is required to fully determine the underlying mechanisms and optimize the use of specific probiotic strains for optimal therapeutic outcomes. Nonetheless, These results emphasize probiotics' potential as a promising adjunctive treatment strategy for UC by targeting macrophage‐mediated inflammation and restoring immune balance in the gut.

## 5 EFFECTS OF PROBIOTICS ON DCS IN UC

Several investigations have shown that alterations in DC distribution or activities are connected with various prevalent diseases.^100^, ^101^, ^102^, ^103^ DCs have the potential to be modulated to reinstate T‐cell tolerance and limit the generation of autoantibodies. Furthermore, it is essential to note that there is variation in functionality within each subgroup of DCs.^100^ Additionally, it has been shown that DCs may affect the development of several disorders, including IBD.^104^, ^105^, ^106^ The pathophysiology of UC is associated with the dysregulation of DC function. Recent studies have provided insights into the capacity of probiotics to regulate DCs, presenting a viable approach for treating UC.^64^, ^65^, ^66^

Okada and colleagues investigated the potential of a newly discovered probiotic yeast strain, Zygosaccharomyces sapae strain I‐6, obtained from Miso, a traditional fermented cuisine in Japan.^64^ The researchers examine the influence of this yeast on distinct immune cells inside the DCs and evaluate its capacity to alleviate inflammation in a murine model of colitis produced by DSS.^64^ The investigators initially picked a single colony of yeast from Miso, mostly based on its ability to induce the synthesis of IL‐10 in CD11c+ bone marrow dendritic cells (BMDCs) in a laboratory setting. IL‐10 is a pivotal cytokine that inhibits inflammation and assumes a critical function in modulating the immune system reaction.^64^ The researchers assessed the anti‐inflammatory properties of strain I‐6 on two types of DCs: CD11c+ BMDCs and a particular subtype known as CD11c+CD103+ DCs. This evaluation was conducted in vitro and in a mouse model, focusing on the mesenteric lymph nodes. The study's findings indicate that the application of strain I‐6 resulted in a notable augmentation in the production of IL‐10 in BMDCs compared to *S. cerevisiae*, a commonly employed yeast strain in probiotic formulations. Significantly, the augmentation in IL‐10 synthesis was facilitated by the activation of immunological receptors known as TLR2 and Dectin‐1.^64^ The study also revealed that β‐glucan derived from strain I‐6, a constituent of the yeast cell wall, elicited elevated levels of IL‐10 production in BMDCs compared to β‐glucan sourced from *S. cerevisiae*. Furthermore, the introduction of strain I‐6 resulted in a significant augmentation in the population of CD11c+CD103+DCs inside the mesenteric lymph nodes.^64^ The primary discovery of this research was that strain I‐6 had significant anti‐inflammatory properties in the mice model of DSS‐induced colitis in comparison to *S. cerevisiae*. Moreover, in the context of colitis, the transplantation of BMDCs treated with strain I‐6 into mice exhibited significant anti‐inflammatory properties, even without oral administration of live I‐6 cells.^64^ In summary, the probiotic yeast strain I‐6 obtained from Miso showed the ability to elicit phenotypic alterations in intestinal DCs. These alterations were characterized by an upregulation in the production of IL‐10, a cytokine known for its anti‐inflammatory properties. Furthermore, strain I‐6 exhibited robust anti‐inflammatory benefits in a mouse model of colitis. This study proposes that fermented foods from Japan, which are deeply rooted in their traditional culture, can potentially be a valuable reservoir of probiotic yeasts. These yeasts might be explored as a possible therapy for IBD and its associated diseases, presenting a prospective avenue for therapeutic intervention in IBD patients.

The study conducted by Lamubol and colleagues focuses on the examination of the possible anti‐inflammatory attributes shown by a specific probiotic strain known as *L. plantarum 22A‐3* (*LP22A3*). The primary objective of the research is to gain insights into the fundamental molecular pathways that contribute to the observed effects of this probiotic strain. The primary emphasis of their investigation lies in assessing the impacts of DSS‐induced colitis on a mouse model.^65^ The TLR2 signaling pathway is often linked to an NF‐κB‐dependent pro‐inflammatory reaction. However, new research suggests that TLR2 signaling differs by cell type and is plastic.^107^ The study conducted by Zhang and colleagues demonstrates that the oral treatment of *L. rhamnosus* leads to an augmentation in intestinal CD103+DCs and an accumulation of mucosal Treg.^108^ Furthermore, Jeon and colleagues demonstrated that CD103+DCs undergo activation via the TLR2/MyD88 pathway, leading to the induction of IL‐10 and IL‐27 synthesis.^109^ It has been shown that the production of TGF‐β1 by lamina propria DCs, which is produced by the collaboration of the TGF‐β‐Smad and TLR2‐AP‐1 signaling pathways, is necessary to develop gastrointestinal Treg.^110^ According to the findings of the Lamubol and colleagues research, it was observed that *LP22A3* possessed the potential to enhance the synthesis of active TGF‐β in the IECs located in the ileum, which is recognized as the terminal segment of the small intestine. Additionally, an elevation in the levels of active TGF‐β was also seen in the serum of mice that were not subjected to DSS treatment.^65^ TGF‐β is a pivotal cytokine that has significant anti‐inflammatory capabilities. In addition, administration of *LP22A3* resulted in increased concentrations of IL‐10 in the circulatory system, which is recognized as a significant anti‐inflammatory mediator.^65^ The mRNA expression levels of genes related to TGF‐β, IL‐10, and Foxp3 (a marker indicative of Tregs) exhibited a particular increase inside the small intestines of mice treated with *LP22A3*. This finding indicates that *LP22A3* has a specific impact on the immune response of the small intestine.^65^ Additionally, the investigation revealed that the administration of *LP22A3* resulted in an augmentation of the mRNA expression of aldehyde dehydrogenase 1 family member A2 (Aldh1a2) and a spike in the number of CD103+DCs inside the small intestine.^65^ DCs expressing the CD103 marker are of significant importance in the control of the immune system. The administration of *LP22A3* stimulated TGF‐β release from the IECs located in the small intestine. This effect is believed to be mediated by the activation of TLR2. Additionally, *LP22A3* facilitated retinoic acid synthesis, a compound renowned for modulating immunological responses. The administration of *LP22A3* therapy led to a notable augmentation in the population of CD103+DCs as well as Foxp3+Tregs.^65^ Both of these cellular phenotypes are recognized for their capacity to produce substantial quantities of anti‐inflammatory cytokines, such as TGF‐β and IL‐10. This phenomenon establishes a protective milieu inside the intestinal tract, reducing its vulnerability to inflammatory processes.^65^ In conclusion, the research indicates that the oral administration of *LP22A3* might serve as a viable treatment approach for IBD owing to its capacity to stimulate the production of TGF‐β, enhance the presence of anti‐inflammatory CD103+DCs, and facilitate the development of Foxp3+Tregs. The results above underscore the potential of *LP22A3* as a favorable probiotic for persons suffering from IBD.

The study conducted by Li and colleagues aimed to examine the influence of commensal intestinal bacteria on the regulation of host immunological tolerance, with a specific emphasis on their possible therapeutic implications for IBDs.^66^ The researchers used a murine model of colitis produced by DSS and implemented several antibiotic interventions to identify distinct bacterial strains and metabolites implicated in immune modulation.^66^ The researchers made a significant finding during their experimental investigations. They observed that *Enterobacter ludwigii*, a microorganism that was present in high quantities in the microbiota of mice treated with the antibiotic metronidazole, had both preventative and therapeutic properties concerning DSS‐induced colitis. Significantly, these consequences were detected despite the coexistence of intricate gut bacteria with *E. ludwigii*.^66^ The interplay between commensal bacteria and immune cells is crucial in maintaining intestinal homeostasis. Nevertheless, a limited number of bacteria have been shown to directly interact with intestinal immune cells or epithelial cells to promote immunological tolerance. The activation of noninflammatory Th17 cells by segmented filamentous bacteria contributes to maintaining gut homeostasis.^111^ One example of this phenomenon is the ability of *Bacteroides fragilis* to directly stimulate the development of Tregs that produce IL‐10 inside the gastrointestinal tract via the activation of TLR2.^112^ The findings of Li and colleagues demonstrated that *E. ludwigii* stimulated the activation of CD103+DCs and Tregs, both of which are crucial in maintaining immunological tolerance and facilitating the resolution of colitis.^66^ A combination of in vitro and in vivo tests verified the immune‐modulating impact. Previous studies have shown that choline has been observed to impact the remission of DSS‐induced colitis.^113^ The beneficial effects of choline on embryonic neurodevelopment and its potential role in preventing cognitive decline have been well recognized. Consequently, clinical studies have assessed choline supplementation's efficacy in treating neurological disorders.^114^ A notable finding of this research was the recognition of choline, a metabolite synthesized by *E. ludwigii*. The research findings indicate that choline can augment the immunological tolerance of dendritic cells, hence facilitating the development of Tregs.^66^ This mechanism proposes that choline plays a significant function in the control of the immune system. The researchers also discovered the molecular mechanism via which *E. ludwigii* induces immunological tolerance in dendritic cells. The researchers discovered that the process included the activation of a7nAChR, which subsequently increased the synthesis of retinoic acid and TGF‐β in DCs. Both RA and TGF‐β have been identified as crucial elements establishing and sustaining immunological tolerance.^66^ In summary, the findings of this research suggest that *E. ludwigii* has a notable protective effect against DSS‐induced colitis in mice. This effect is likely mediated by choline and the activation of RA and TGF‐β in dendritic cells through a7nAChR. The results indicate possible treatment strategies for IBDs, emphasizing the significance of specific commensal bacteria and their metabolites in regulating the immune response inside the gastrointestinal tract. The aforementioned observations may have significant ramifications for the development of innovative therapies for individuals suffering from IBD. Collectively, these studies underscore the potential of probiotics and certain commensal bacteria to modulate immune responses, offering promising opportunities for advancing therapeutics for IBD. A deeper comprehension of the interplay between these microbes and the immune system. reveals new possibilities for the management and therapy of patients with IBD, offering possible improvements to their general health and quality of life.

## 6 EFFECTS OF PROBIOTICS ON T CELLS IN UC

Multiple investigations have shown probiotics to modulate T cells, which are essential for the immunological response.^115^, ^116^, ^117^ T‐lymphocyte subset dysregulation has recently been recognized as a key contributor to the occurrence of UC.^118^, ^119^, ^120^ In this section, we explore the potential of probiotics to influence T‐cell responses. A groundbreaking study by Di Giacinto and colleagues revealed that administering probiotics (specifically VSL#3) to mice during the remission period between two episodes of colitis resulted in a milder form of recurrent colitis compared to mice given a placebo.^80^ It was determined that probiotics' influence on the lamina propria mononuclear cells population was responsible for the observed beneficial impact. The administration of probiotics boosted the generation of IL‐10 and the proportion of regulatory CD4+T cells (also known as LAP+T cells) expressing surface TGF‐β in the form of latency‐associated protein.^80^ The presence of LAP+T cells was reliant on IL‐10 production, as blocking the IL‐10 receptor hindered their appearance. Furthermore, LAP+T cells played a crucial role in the protective effect of probiotics, as blocking the IL‐10 receptor, inhibiting TGF‐β, or depleting LAP+T cells abolished their ability to confer protection to naive recipients.^80^ These findings suggest that probiotics, particularly VSL#3, have the potential to modulate the immune system and promote regulatory responses that mitigate inflammation and enhance intestinal well‐being. Understanding the biological processes through which probiotics exercise their positive effects provides important insights for creating novel treatment techniques to treat IBD, such as those targeting regulatory cells that express IL‐10 and TGF‐β.

In a different investigation that Zhao and colleagues carried out, it was demonstrated that administering a combination of probiotics (Bifico capsules) effectively lessened the severity of experimental colitis in mice.^68^ The treatment resulted in decreased colonic weight and colonic weight index compared to untreated mice with colitis.^68^ Moreover, the mice treated with probiotics exhibited improved colon length and a lower percentage of body weight gain.^68^ The probiotics treatment also increased the number of regulatory T cells inside the MLN, namely CD4+CD25+Foxp3+ T cells. Furthermore, the expression of pro‐inflammatory cytokines such as TNF‐α and IFN‐γ was reduced, while the synthesis of anti‐inflammatory cytokines IL‐2, IL‐4, and IL‐10 was increased in the colonic tissues of mice treated with probiotics.^68^ These results may have significant repercussions for the possible therapeutic use of probiotics in treating colitis. By enhancing the presence of Tregs and modulating the balance of cytokines, probiotics can promote immune regulation and reduce inflammation in the colon. This study further supports the advantages of probiotics concerning IBD and underscores their potential as a complementary treatment strategy.^68^

Qu and colleagues conducted new research to evaluate the therapeutic efficacy of certain strains of *B. bifidum* for managing chronic colitis. The focus of the study was primarily on controlling and preventing colitis in the setting of CD.^69^ The impact of FJSWX19M5 treatment on the histology of the colonic mucosa is vital because achieving endoscopic mucosal healing is often regarded as the most reliable indicator of long‐term remission in the clinical arena of CD therapy.^121^ The observed protective benefits of some probiotics may be partially attributable to their ability to regulate the function of the intestinal barrier, particularly the tight junction (TJ) function and the formation of mucin. In their study, Qu and colleagues demonstrated that the particular strain of *B. bifidum*, namely *FJSWX19M5*, could enhance TJ's development and the integrity of the colonic physical barrier in mice treated with TNBS. These findings align with previous results on the subject matter.^122^, ^123^ During the tests, significant favorable effects were observed in the *B. bifidum* strain FJSWX19M5. The intervention provided relief from body weight loss, mitigated colonic shortening and damage, reduced gut inflammation indicators, and restored colonic metabolism equilibrium in mice afflicted with TNBS‐induced chronic colitis.^69^ The bioactive compounds originating from symbiotic microbiota are crucial in forming the intestinal immune system and controlling IL‐10 release.^124^

It is possible to stimulate the production of IL‐10 in Foxp3+ cells. Treg cells are paramount in establishing immune tolerance towards antigens on mucosal surfaces, effectively avoiding inflammation in the gastrointestinal tract.^125^ The study conducted by Qu and colleagues provided evidence that the administration of *FJSWX19M5* resulted in the augmentation of Tregs and had a role in the recovery of the intestinal barrier.^69^ This observation implies that *FJSWX19M5* could possess immunomodulatory characteristics that contribute to regulating the immune response inside the gastrointestinal tract. Additionally, it was shown that the co‐cultivation of *B. bifidum* strains with anticolitis capabilities induced an elevation in the expression of the cytokine that inhibits inflammation IL‐10 and a decreased ratio of pro‐inflammatory cytokines (IL‐22/IL‐10 and IL‐17/IL‐10) as compared to nonprotective strains. This implies that the advantageous strains have the potential to regulate the immune response in a manner that decreases inflammation associated with colitis.^69^ Furthermore, the findings of Qu and colleagues showed that the injection of heat‐killed *FJSWX19M5* positively impacted symptoms associated with colitis. This suggests that the therapeutic effects of this treatment may not only depend on the presence of live bacteria.^69^ This implies that strains of *B. bifidum* or their lysates have the potential to be investigated as therapeutic options for the treatment of CD. In conclusion, the research posits that some strains of *B. bifidum*, namely FJSWX19M5, have promising capabilities in alleviating chronic colitis via the enhancement of gut barrier function, modulation of the immunological response, and restoration of gut microbiota equilibrium. The aforementioned results provide encouraging perspectives on the use of probiotics as a prospective therapeutic strategy for the management of CD and its associated disorders.

The study by Huang and colleagues aimed to examine the immunoregulatory function of *L. paracasei R3* (*L.p R3*) in a mouse model of colitis caused by DSS.^70^ The researchers isolated the strain *L. paracasei*, identified explicitly as *L.p R3*, from newborn feces. This particular strain was selected based on its notable capacity to form biofilms, which holds promise for augmenting its resilience and efficacy inside the gastrointestinal tract.^70^ *L.p*, a Gram‐positive bacteria, has significance as a prominent constituent of the *Lactobacillus* genus. It is often seen in several human anatomical sites, such as the oral cavity, gastrointestinal tract, and fermented food products. *L.p* can modulate gut microbiota equilibrium, bolster human immune response, and exhibit antitumor properties.^126^, ^127^ Certain *Lactobacillus* species have been proven to have anti‐inflammatory properties in IBD disorders.^128^ According to the research conducted by Huang and colleagues, it was determined that *L.p R3* had a beneficial effect on mice suffering from colitis produced by DSS. More precisely, it improved the overall manifestations of colitis, including symptoms such as diarrhea, loss of weight, and bleeding from the rectum.^70^ Additionally, it was shown that *L.p R3* exhibited a reduction in the infiltration of inflammatory cells into the intestinal tissue of mice afflicted with colitis. This finding implies that the strain has anti‐inflammatory capabilities and may contribute to mitigating the immunological response linked to colitis.^70^ Th17 refers to a distinct population of T cells that can produce IL‐17A. IL‐17A is a cytokine known for its pro‐inflammatory properties, exhibiting diverse functions across numerous disease contexts.

In comparison to persons without any health conditions, the levels of IL‐17A were shown to be considerably elevated in patients diagnosed with UC.^129^, ^130^ The research conducted by Huang and colleagues exhibited that the administration of *L.p R3* decreased the infiltration of inflammatory cells inside the intestinal tissue of mice afflicted with colitis. This implies that the strain has anti‐inflammatory capabilities and can potentially mitigate the immunological response linked to colitis.^70^ The findings of this investigation demonstrated that *L.p R3* had a dual impact on the immune system. The compound inhibited the development process of Th17 cells, a subset of immune cells known for their pro‐inflammatory properties. Conversely, it exhibited a stimulatory effect on the functionality of Treg cells, a distinct subset of immune cells responsible for immunological regulation.^70^ The regulation of the immune response plays a vital role in maintaining harmonized and regulated immune responses inside the gastrointestinal tract. The results of this investigation indicate that *L.p R3* exhibits noteworthy therapeutic efficacy in mitigating symptoms of colitis and pathological harm in murine subjects. This is accomplished by the modulation of the immune response, especially by modulating the equilibrium between pro‐inflammatory Th17 cells and anti‐inflammatory Treg cells in colitis produced by DSS. In conclusion, our research emphasizes the potential of *L. pR3* as a viable probiotic option for the treatment of colitis, specifically within the framework of IBD. The possibility that this substance alleviates inflammation and modulates the equilibrium of immune cell populations indicates its potential involvement in alleviating the symptoms and pathology related to colitis in murine models. The results above highlight the significance of probiotics in the field of IBD research and their potential as therapeutic agents for associated disorders in humans.

The research undertaken by Park and colleagues sought to investigate the possible therapeutic benefits of *L. acidophilus* in the context of acute colitis using a mouse model.^71^ Limited research exists about the impact of *L. acidophilus* on the regulation of Th17 and Treg cells. In a mouse model of allergies, *L. acidophilus* had a mitigating effect on blood levels of IL‐6 and IL‐17, as well as a downregulating impact on the expression of IL‐17A and retinoic acid‐related orphan receptor (ROR)‐γt (RORγt) in the spleen.^131^ It has been demonstrated in recent research that oral treatment of *L. acidophilus* efficiently reduced the production of IL‐17, TNF‐α, and IL‐23, as well as STAT3 and phosphorylated STAT3, in the colon tissue of a colitis model created by DSS.^132^ The study revealed that the administration of *L. acidophilus* increased the proliferation of Treg cells inside the intestine intraepithelial and lamina propria lymphocytes (LPLs) in a TNBS model.^133^ In the murine allergy paradigm, the spleen of the *L. acidophilus*‐treated group exhibited elevated expression levels of CD25, Foxp3, and TGF‐β.^131^ A study conducted by Park and colleagues provided evidence supporting the efficacy of *L. acidophilus* medication in mitigating the severity of colitis produced by DSS.^71^ This finding indicates that *L. acidophilus* may have a preventive function in attenuating the inflammatory response often associated with colitis. Furthermore, the study's results indicate that the application of *L. acidophilus* led to the suppression of inflammatory mediators, such as TNF‐α, IL‐6, IL‐1β, and IL‐17, inside the colon tissues. The production of these cytokines is often attributed to Th17 cells and is closely linked to the process of inflammation.^71^ The inhibition of these cytokines signifies a decrease in the inflammatory response. The application of *L. acidophilus* in an in vitro setting resulted in the stimulation of Tregs and an augmentation in the synthesis of the anti‐inflammatory cytokine IL‐10.^119^ This finding indicates that *L. acidophilus* has the capacity to enhance immunological modulation and mitigate the pro‐inflammatory response, hence augmenting its therapeutic efficacy. In addition, the investigation also noted a decrease in the levels of α‐smooth muscle actin, which serves as an indicator of activated myofibroblasts, as well as type I collagen, after the administration of *L. acidophilus* therapy. This finding suggests that *L. acidophilus* may potentially play a significant role in the prevention or mitigation of fibrosis progression in the colon, a prevalent consequence associated with IBD. In summary, the findings of this research indicate that *L. acidophilus* exhibits promise as an innovative therapeutic approach for IBD. The therapeutic actions of this substance are ascribed to its capacity to regulate the equilibrium between anti‐inflammatory Treg cells and pro‐inflammatory Th17 cells, reduce the rate of fibrosis development in the colon and prevent the generation of pro‐inflammatory cytokines. The findings presented above demonstrate the potential effectiveness of *L. acidophilus* as a probiotic in the treatment and management of IBD, particularly in instances of acute colitis.

The research carried out by Jia and colleagues sought to investigate the effects of *Porphyromonas gingivalis* (*Pg*), a pathogenic bacteria, and *L. rhamnosus GG* (*LGG*) on the modulation of Th17 and Treg cell equilibrium within CD4+T cells.^72^ Maintaining this equilibrium is vital in regulating inflammation, especially in immune‐inflammatory disorders like IBD. The relationship between Pg and TLR2 and TLR4 has been extensively investigated because of the diverse lipid A structure shown by Pg. TLR2 mainly recognizes lipopeptides and lipoteichoic acid from gram‐positive bacteria, whereas the endotoxin LPS from gram‐negative bacteria is primarily detected by TLR4.^134^ In a recent research, a specific subset of CD4+T cells known as Treg17 cells was found. These cells can produce both Foxp3 and IL‐17, and this expression may vary depending on the level of inflammation present. The IL‐17‐producing Tregs discussed in this study are derived from the CD4+T‐cell subset that TGF‐β and IL‐6 stimulate in an in vitro setting. These Treg cells express the transcription factor RORγt and produce IL‐10.^135^ The shift from generic Th17 cells to Treg cells is influenced by three key determinants: particular transcription factors, cytokines, and TLRs.^136^ Jia and colleagues demonstrated that Pg can initiate apoptosis, or programmed cell death, in CD4+T cells.^72^ The noticed result was an increase in the expression of RoRt, which has been demonstrated to contribute to the development and function of Th17 cells. Additionally, there was an increase in the secretion of pro‐inflammatory mediators, including IL‐17 and IL‐6.^72^ Significantly, the administration of *Pg* extract resulted in the downregulation of Foxp3, a crucial transcription factor necessary for Tregs, as well as a reduction in the production of IL‐10 and TGF‐β. The observed effects were facilitated via the TLR4 pathway.^72^ On the other hand, the administration of *LGG* extract contributed to preserving Th17/Treg equilibrium. The intervention led to a reduction in the proportion of IL‐17+Th17 cells and an upsurge in the proportion of CD25+ Foxp3+ Treg cells. TLR2 was found to be the mechanism responsible for the effect observed.^72^ In a colitis model experiment generated by DSS, CD4+T cells driven by *Pg* exacerbated the colitis condition by elevating the Th17/Treg cells ratio in both the colon and LPLs.^72^ In contrast, the co‐administration of *Pg* and *LGG*‐stimulated CD4+T cells mitigated colitis by reducing the Th17/Treg ratio. This observed outcome was facilitated via the JAK‐STAT signaling pathway.^72^

This work provides evidence that pathogenic bacteria, such as Pg, and probiotic bacteria, such as *LGG*, can directly impact the equilibrium between Th17 and Treg cells through TLRs. Pg induces inflammation by accelerating the expansion of Th17 cells and suppressing the activity of Treg cells via the TLR4 route. Conversely, *LGG* maintains the balance between Th17 and Treg by modulating the TLR2 pathway. The results above emphasize the possibility of therapies using microorganisms to regulate the immune response, offering hope in comprehending and perhaps managing immuno‐inflammatory disorders such as IBD.

The study conducted by Jeong and colleagues examines the possible therapeutic properties of *L. plantarum C29* in the setting of colitis produced by TNBS in mice. The research explicitly explores this probiotic's influence on the equilibrium between Th17 cells and Tregs and the involvement of NF‐κB activation in this process.^73^ This study aims to examine the possibility of *L. plantarum C29* in rectifying the imbalance between Th17 and Treg cells in mice with colitis produced by TNBS. Additionally, this study seeks to gain insights into the processes behind this therapeutic effect. The results of this investigation showed that administering *L. plantarum C29* effectively impeded the process of splenic T cell differentiation into Th17 cells. Furthermore, it was observed that the expression of RORγt and IL‐17 was significantly reduced in vitro as a result of this therapy.^73^ In contrast, the researchers suggested that the administration of *C29* therapy facilitated the process of Treg differentiation, which involves the development of regulatory immune cells.^73^ The administration of *L. plantarum C29* via oral route to mice afflicted with colitis induced by TNBS led to various beneficial effects. These effects encompassed the mitigation of colon shortening, which indicates reduced inflammation and the reduction in MPO activity, demonstrating a decline in neutrophil infiltration. Moreover, the iNOS and COX‐2 expression in the colon was downregulated, and the activation of NF‐κB, a crucial transcription factor implicated in inflammation, was blocked in the colonic tissues of the mice.^73^ The administration of *C29* therapy led to decreased inflammatory mediators inside the colon, including TNF‐α, IL‐17, and IL‐1β. Furthermore, it resulted in an augmentation in the IL‐10 production. According to Jeong and colleagues research, *C29* significantly reduced the development of Th17 cells in the colon, which TNBS caused. Additionally, *C29* treatment resulted in a decrease in the production of IL‐17 and RORγt, both of which are closely related to Th17 cells.^73^

On the other hand, the administration of *C29* therapy enhanced the differentiation of Tregs in the colon. The upregulation of IL‐10 and forkhead box P3 (Foxp3) was observed, both of which are closely linked to Tregs.^73^ The research findings indicate that *L. plantarum C29* has promising therapeutic properties in mitigating TNBS‐induced colitis in mice. The outcome above is attained by using many strategies, such as suppressing Th17 cell development, facilitating Treg cell differentiation, and regulating NF‐κB activation. The ultimate result is a restoration of equilibrium in the Th17/Treg ratio, characterized by a decrease in cytokines that promote inflammation and an augmentation of anti‐inflammatory mediators. The findings of this investigation suggest that *C29* might possess potential therapeutic benefits in treating colitis and other inflammatory disorders. This might be attributed to its ability to modulate the immune response and mitigate inflammation via regulating NF‐κB.

Ogita and colleagues investigate the effects of *Streptococcus thermophilus ST28* (*ST28*) on the generation of cytokines and its possible anti‐inflammatory capabilities in a model of DSS‐induced colitis.^74^ The research evaluated the influence of *S. thermophilus ST28* on the production of cytokines by splenocytes from mice that were stimulated with a combination of TGF‐β and IL‐6.^74^ It has been discovered that these cytokines have a role in promoting the growth of Th17 cells, a process linked to inflammation. The experimental findings indicate that *ST28* had dual effects, suppressing both IL‐17 production and the population of Th17 cells in the activated splenocytes. This finding provides more evidence for the proposition that *ST28* has the capacity to impede the differentiation or proliferation of Th17 cells, which have been implicated in the pathogenesis of autoimmune and inflammatory disorders.^74^ The present research aimed to assess the anti‐inflammatory properties of *ST28* in DSS‐induced colitis. The administration of *ST28* by oral route demonstrated a significant improvement in the intestinal lesions caused by DSS, indicating a potential protective action against colitis’^74^ Following treatment with DSS, there was an observed increase in IL‐17 production in LPLs, suggesting the occurrence of an inflammatory response inside the intestinal region. Nevertheless, the treatment of *ST28* resulted in a significant reduction in the production of IL‐17 in LPLs, indicating its potential to alleviate inflammation in the gastrointestinal tract.^74^ The administration of *ST28* therapy reduced the proportion of Th17 cells within the LPLs of mice with colitis caused by DSS's^74^ This suggests that *ST28* may modulate the immune response in the gut by inhibiting the expansion or activation of Th17 cells. In conclusion, the findings of this research provide evidence that *S. thermophilus ST28* has anti‐inflammatory characteristics, namely by inhibiting the generation of IL‐17 and lowering the number of Th17 cells. The involvement of Th17 cells in inflammatory conditions, such as IBD, indicates that *ST28* might potentially be utilized as a medicinal agent in treating disorders mediated by Th17 cells, especially in the setting of IBD. This study enhances the comprehension of probiotics as prospective therapies for immune‐mediated diseases.

The study conducted by Yan and colleagues explores the potential therapeutic benefits of *Bacteroides uniformis* (*Bu*) and its metabolites in a colitis model caused by DSS's^75^ Prior research has shown that extended administration of *Bu CECT 7771* to mice may induce changes in the makeup of gut microbiota and facilitate the growth of potentially beneficial bacterial species.^137^, ^138^ The administration of *BCECT 7771* has been shown to augment the functionality of macrophages and DCs, reinstating the antigen recognition capacity of DCs and promoting the expansion of T lymphocytes.^137^ This investigation's findings demonstrated that Bu's administration effectively mitigated the advancement of colitis, indicating its potential protective efficacy in the DSS‐mouse model.^75^ Additionally, the *Bu* therapy has shown the ability to reinstate the production of mechanical and immunological barrier proteins, which are often disrupted in cases of colitis.^75^ This suggests that *Bu* might help preserve the intestinal barrier's integrity. Furthermore, *Bu* therapy was linked to a rise in the number of good bacteria such as *Bifidobacterium* and *Lactobacillus vaginalis* while decreasing the prevalence of pathogenic microbes and reducing the abundance of pathogenic *Escherichia‐Shigella*.^75^ This implies that Bu has the potential to benefit the makeup of the gut microbiota. Yan and colleagues findings demonstrated that Bu can regulate the metabolism of bile acids in the intestines. It controlled important metabolites such as hyodeoxycholic, isolithocholic acid, and alpha‐muricholic.^75^

Bile acids are involved in many physiological activities, including immunological control. Furthermore, the findings of this research indicate that *Bu* exerted a notable influence on the modulation of essential regulatory proteins involved in the NF‐κB and mitogen‐activated protein kinase signaling pathways in colonic tissues.^75^ Furthermore, it impacted the differentiation process of TH17 cells, which have been linked to inflammatory responses. Nevertheless, *Bu* did not directly impede the development of TH17 cells in a laboratory setting. Instead, it seemed to influence this process inside the lamina propria, indicating a multifaceted mechanism.^75^ The results suggest that *Bu* or bile acid supplements may have promise as therapeutic interventions for colitis and other conditions characterized by impaired intestinal barrier function. The potential therapeutic efficacy of *Bu* is attributed to its impact on bile acid metabolism, gut microbiota, and immunological modulation. In conclusion, our work elucidates the diverse effects of *Bu* in mitigating colitis in a murine model. The factors above impact the makeup of gut microbiota, the metabolism of bile acids, and the immunological response, namely the differentiation of TH17 cells. The findings of this investigation suggest that treatments using *Bu* or bile acids show potential as therapeutic approaches for colitis and other illnesses defined by a malfunction in the intestinal barrier. This study enhances our comprehension of the function of certain gut microorganisms and metabolites concerning gastrointestinal well‐being and pathological conditions.

The research carried out by Zou and colleagues sought to investigate the potential therapeutic benefits of *B. infantis* in the context of experimental colitis in mice. Specifically, the researchers focused on examining the influence of *B. infantis* on T‐cell subsets and cytokine profiles.^76^ The study's primary aim is to understand how *B. infantis* affects T cell subsets and related cytokine responses and whether it can mitigate the severity of TNBS‐induced colitis in mice.^76^ The findings of this investigation indicate that the administration of a modest dosage of *B. infantis* did not result in noteworthy alterations in cytokine profiles in mice with normal physiological conditions. In contrast, the administration of a high dosage of *B. infantis* resulted in an upregulation of IL‐12p40, IL‐2, IFN‐γ as a Th1 cytokines, IL‐23, RORγt, IL‐21 as a Th17 cytokines and transcription factor, as well as IL‐10, Foxp3 as a regulatory Treg‐related cytokines and transcription factor in mice with normal immune function.^76^ This finding suggests that administering a high dosage of *B. infantis* can regulate the immune response by influencing the activity of specific cytokines and transcription factors. The utilization of flow cytometry research demonstrated that *B. infantis* led to an augmentation in the quantities of CD4+Foxp3+Tregs and Th17 cells in the mesenteric lymph nodes (MLNs). This implies that *B. infantis* can impact the equilibrium of these T cell subsets, which perform a vital function in immunological modulation and inflammation.^76^ The discovery, as mentioned above, implies that the administration of a substantial dose of *B. infantis* can modulate the immune response via its influence on the activity of specific cytokines and transcription factors. Flow cytometry in this investigation unveiled that the presence of *B. infantis* augmented the quantities of CD4+Foxp3+Tregs and Th17 cells within the MLNs. This suggests that *B. infanti*s has the ability to influence the balance of these T cell subsets, which have a significant function in regulating the immune system and inflammation.

Liu and colleagues displayed that the probiotic combination VSL#3 reduces colitis induced by DSS in mice by inhibiting Tfh cells, which are responsible for inflammation.^139^ The researchers utilized a DSS‐induced mice model of colitis and administered VSL#3, a mixture of eight probiotic bacteria, to the mice for a duration of 60 days, which resulted in improved disease outcomes.^139^ Treatment with VSL#3 reduced MPO activity, histological activity index (HAI), and disease activity index (DAI), indicating decreased colonic inflammation.^139^ Additionally, VSL#3 reversed the disrupted humoral immunity observed in colitis, as evidenced by decreased levels of immunoglobulins (including IgM, IgA, and IgG) in the colonic mucus. Furthermore, VSL#3 treatment reduced the number of Tfh cells in the MLN.^139^ This study highlights the advantageous consequences of VSL#3, a probiotic mixture, in the context of colitis. These results indicate that VSL#3 attenuates DSS‐induced colitis by suppressing Tfh cells that play a role in autoimmunity. By modulating the Tfh cell population, VSL#3 exerts anti‐inflammatory effects, improving disease outcomes. The reduction in colonic inflammation, as indicated by lower DAI, HAI, and MPO activity, underscores the therapeutic potential of VSL#3 in treating IBD.^139^ Moreover, this study explains how Tfh cells contribute to the progression of IBD. The downregulation of Tfh cells by VSL#3 suggests that targeting these cells might have promise as a treatment strategy for IBD. These findings provide valuable insights into the underlying mechanisms of probiotic action and open up avenues for further research and the establishment of specialized treatments for IBD.^139^ The study suggests that VSL#3 administration exerts anti‐inflammatory effects in DSS‐induced colitis, partially mediated by suppressing Tfh cells. These discoveries advance the knowledge of the immunomodulatory properties of probiotics and their potential use as adjunctive therapies for IBD.

Kim and colleagues performed research in which they discovered that *Lactobacillus plantarum CBT LP3 (LP3)* has a beneficial impact on colitis by modulating T cells in mice.^79^ The investigators employed a DSS‐induced colitis mouse model and randomly allocated animals to one of three groups: control, DSS‐treated with oral vehicle administration, and DSS‐treated with oral administration daily of *LP3* for 7 days after DSS administration. Disease activity, histology, immune cell subsets, gene expression levels, and cytokine profiles were all studied concerning *LP3*.^79^ When *LP3* was administered, there was a dramatically reduced disease progression and improved histopathology compared to the control group. *LP3* exhibited anti‐inflammatory properties, as demonstrated by an increase in the population of Tregs and Th2 cells in splenocytes.^79^ Furthermore, *LP3* administration restored the presence of goblet cells, responsible for mucus production, and suppressed the expression of pro‐inflammatory cytokines. These findings indicate that *LP3* acts as an immunomodulator, regulating T‐cell responses and creating an anti‐inflammatory environment in the colon.^79^ This study highlights the potential therapeutic significance of *L. plantarum CBT LP3* in treating colitis. The findings suggest that *LP3* possesses anti‐inflammatory effects and ameliorates colitis by modulating T‐cell responses. The induction of Tregs and Th2 cells by *LP3* indicates its role in immune regulation, promoting an immune balance that favors the resolution of inflammation.^79^ The ability of *LP3* to restore goblet cells and suppress pro‐inflammatory cytokines further supports its therapeutic potential for inflammatory bowel diseases such as colitis. These results advance our knowledge of the processes underpinning the beneficial effects of *LP3* and emphasize its potential as an immunomodulatory compound as a therapy option for IBD.^79^ This study suggests that *L. plantarum* CBT *LP3* exhibits promising immunomodulatory effects and ameliorates colitis by modulating T‐cell responses. These results promote future investigation into using *LP3* as a possible therapeutic tool and provide insightful information for establishing probiotic‐based treatments for IBD.

The probiotic bacteria *Lactobacillus kefiri* is investigated by Curciarello and colleagues in their research to determine how it affects intestinal T cells generated from individuals with active IBD, such as UC and CD.^77^ IBD is characterized by inflammation driven by T cells, making them a prime target for therapeutic strategies.^77^ This study aimed to examine the impact of *L. kefiri* on intestinal T cells isolated from individuals with active IBD.^77^ Surgical samples and mucosal biopsies were collected from IBD patients and healthy donors to isolate LPMC, including T cells residing in the intestinal lining. These lamina propria T cells (LPTC), which are known as entero‐adhesive *E. coli*‐specific cells, have been generated for additional investigation.^77^

The results of this investigation showed various vital results. First, ex vivo inflamed biopsies of *L. kefiri* inhibited the spontaneous production of cytokines that promote inflammation, such as IL‐6 and IL‐8, suggesting its potential to mitigate inflammation in IBD. Moreover, when LPTC from IBD patients were activated and exposed to *L. kefiri*, they exhibited decreased proliferation rates and reduced production of key cytokines associated with inflammation, such as IFN‐γ, TNF‐α, IL‐13, and IL‐6. This indicates that *L. kefiri* can suppress the inflammatory response of T cells in patients with active IBD.^77^ Additionally, the probiotic increased the frequency of CD4+FOXP3+LPTC, which are Tregs associated with immune tolerance, and elevated levels of IL‐10. This proposes that *L. kefiri* promotes the generation of Tregs and enhances IL‐10 production, further dampening the inflammation in IBD.^77^ In conclusion, this work offers the first account of the immunomodulatory impacts of *L. kefiri* CIDCA 8348 on human intestinal cells obtained from IBD patients. By elucidating the mechanisms through which probiotics interact with immune mucosal cells, this research opens up new possibilities for developing novel treatments and preventive strategies for IBD. The results support the practicability of using *L. kefiri* as a therapeutic tool in managing inflammation and promoting immune balance in patients with IBD.^77^

Chua and colleagues have investigated the possible anti‐inflammatory benefits of an engineered probiotic bacterium, *E. coli Nissle 1917* (*EcN*), through interferon‐driven immunomodulation.^78^ The researchers focused on IBD and hypothesized that promoting an interferon response could alleviate inflammation.^78^ To test their hypothesis, the researchers genetically modified EcN to produce and release interferon lambda 1 (IFNL1), a type III interferon, in response to NO, a known marker of colorectal inflammation. They conducted experiments using two in vitro models: a coculture model involving Caco‐2 IEC and Jurkat T cells and a 3D coculture IBD model comprising T cells, IEC, and myofibroblasts.^78^ The investigation findings showed that the modified EcN strains had anti‐inflammatory properties. The *EcN* strains that produce IFNL1 increased the expression of Foxp3, a hallmark for Tregs, in T cells. A reduction in the generation of cytokines that promote inflammation, including IL‐13 and IL‐33, was caused by this increase, effectively mitigating inflammation in both in vitro models.^78^ Additionally, the modified strains protected the barrier function of the intestinal lining even under inflammatory circumstances by maintaining epithelial cell layer integrity in an inflammatory environment. Therapy with IFNL1‐expressing *EcN* in the 3D coculture model raised the number of Tregs and boosted the generation of the L‐10.^78^ Overall, the present study elucidated the anti‐inflammatory properties of probiotics expressing IFNL1 in two in vitro models of IBD. The findings suggest the potential of utilizing live biotherapeutics, such as engineered probiotics, for immunotherapy in IBD. By modulating the immune response and reducing inflammation, IFNL1‐expressing probiotics offer a promising approach to the designing innovative therapies for IBD patients. This study contributes to the advancement of knowledge of the immunomodulatory properties of genetically modified probiotics, highlighting their potential as therapeutic agents in the area of IBD research and therapy.

## 7 CONCLUSION

In conclusion, the review emphasizes the significance of probiotics in regulating immune cells in UC. The available research indicates that probiotics can potentially mitigate symptoms of UC via the modulation of immune response and the attenuation of gut inflammation. Some different mechanisms, including the stimulation of Treg cells, enhancement of the performance of the intestinal barrier, and inhibition of pro‐inflammatory cytokines, have explained the immune‐modulating properties of probiotics. For example, Jang and colleagues indicated that VSL#3 reduces the severity of colitis, infiltration of colonic macrophages, and levels of cytokines in the bloodstream.^21^ Nevertheless, it does not reduce the inflammatory effects of M1 macrophages. A further study discovered that *Lp082* reduces neutrophil infiltration, protects the mucosal barrier, effectively modifies the gut flora, and has therapeutic impacts on colitis in mice.^82^ While probiotics show promise in managing UC, several challenges must be addressed to optimize their therapeutic potential. Future research should focus on identifying the most effective probiotic strains, determining optimal dosage regimens, and developing targeted delivery systems to enhance their efficacy.

Furthermore, more investigation is necessary to gain an in‐depth knowledge of the underlying mechanisms through which probiotics modulate UC immune cells. Further comprehensive clinical studies are essential to assess probiotics' long‐term safety and effectiveness in treating UC. By addressing these challenges, probiotics can emerge as a promising treatment option for UC patients. They may also reduce the reliance on long‐term use of immunosuppressive drugs, thereby minimizing potential side effects. Incorporating probiotics into the treatment regimen can improve therapeutic outcomes, enhance quality of life, and potentially decrease disease relapse rates in individuals with UC. More investigation is required to identify specific probiotic strains that exhibit superior efficacy in managing UC. Comparative trials that directly compare different strains can offer valuable insights into their unique effects and help customize probiotic interventions for individual patients. Understanding the precise mechanisms through which probiotics exert their therapeutic effects in UC is crucial. Exploring the interactions between the host immune system, probiotics, and the gut microbiota can provide a more profound comprehension of these mechanisms and guide the establishment of more targeted probiotic therapies. Furthermore, investigating the potential synergistic effects of combining probiotics with other therapeutic approaches, such as dietary modifications, prebiotics, or specific medications, should be a focus of future research. Such combination therapies may enhance overall treatment efficacy and provide a more comprehensive management strategy for UC. Overall, the review highlights the potential of probiotics as a safe and effective therapeutic approach for UC. However, further investigation is required to determine the appropriate dose, duration, and strain of probiotics for treating UC. Additionally, research is needed to understand further the underlying processes through which probiotics modulate immune cells in the gut. This understanding could pave the way for developing more targeted and personalized probiotic therapies for UC.

## AUTHOR CONTRIBUTIONS

**Ni Guo**: Conceptualization; visualization; writing—original draft. **Lu‐lu Lv**: Project administration; writing—review & editing.

## CONFLICT OF INTEREST STATEMENT

The authors declare no conflict of interest.
